# The Amyloid Precursor Protein Intracellular Domain-Fe65 Multiprotein Complexes: A Challenge to the Amyloid Hypothesis for Alzheimer's Disease?

**DOI:** 10.1155/2012/353145

**Published:** 2012-02-09

**Authors:** Daniel A. Bórquez, Christian González-Billault

**Affiliations:** Laboratory of Cell and Neuronal Dynamics, Department of Biology and Institute for Cell Dynamics and Biotechnology (ICDB), Faculty of Sciences, Universidad de Chile, Santiago 7800024, Chile

## Abstract

Since its proposal in 1994, the amyloid cascade hypothesis has prevailed as the mainstream research subject on the molecular mechanisms leading to the Alzheimer's disease (AD). Most of the field had been historically based on the role of the different forms of aggregation of *β*-amyloid peptide (A*β*). However, a soluble intracellular fragment termed amyloid precursor protein (APP) intracellular domain (AICD) is produced in conjunction with A*β* fragments. This peptide had been shown to be highly toxic in both culture neurons and transgenic mice models. With the advent of this new toxic fragment, the centerpiece for the ethiology of the disease may be changed. This paper discusses the potential role of multiprotein complexes between the AICD and its adapter protein Fe65 and how this could be a potentially important new agent in the neurodegeneration observed in the AD.

## 1. Introduction

The APP is a type I transmembrane protein with characteristics of an orphan receptor, which shares with other members of its class a particular signaling mechanism termed regulated intramembrane proteolysis (RIP) [1].

RIP requires that the transmembrane protein undergoes two consecutive cleavage events. The first occurs outside the transmembrane domain, usually in response to ligand binding, inducing the release of the extracellular domain. This first cleavage event elicits a conformational change that triggers the second proteolytic cleavage which takes place on the transmembrane segment. The intracellular cytoplasmic fragment released translocates to the nucleus where it activates gene transcription [1]. This mechanism controls several cellular processes, such as the unfolded protein response [2], cholesterol synthesis [3], and cell fate instruction [4].

RIP of the APP is mediated by three different proteases. While *α*- and *β*-secretases catalyze extracellular cleavage, the *γ*-secretase complex cuts at the intramembrane domain and leads to the generation of two peptides: an APP active fragment, termed AICD and the A*β* [1]. The stoichiometry of both AICD and A*β* fragments has been a controversial issue. One study shows that the absence of the *β*-secretase does not affect AICD production [5]. In contrast, two independent groups indicate that AICD is produced mainly from the 695 aminoacids isoform of APP through the amyloidogenic pathway (dependent on *β*-secretase activity) [6, 7] and is therefore generated in equimolar quantities with A*β* [8]. The last one accumulation and the formation of various aggregates and deposits in the brain have been the main hypothesis to explain the neuropathological development of AD for almost 20 years [9]. Initially, the study of the functions associated with the AICD was limited by the hindrance in its detection [10]. However, recent studies showing that the levels of the AICD are increased in brains of AD patients and murine models reproducing the disease [11], open up the possibility that this fragment participates in the molecular mechanisms contributing to AD.

## 2. The AICD Interactome: Functions and Dysfunctions in the Route to AD

The AICD is the most evolutionarily-conserved region of the APP, accounting for its functional importance. Despite its relatively small size (59 aminoacids or less), it acts as a docking site for a particularly large group of intracellular proteins. Amongst this group of proteins are Pin1 [12], the X11 protein family [13], disabled (Dab)-1 [14], Shc [15], JNK-interacting protein (JIP)-1 [16], and the Fe65 protein family [17–19], which includes Fe65 itself and two closely related homologues, Fe65L1 and Fe65L2. Fe65 family members contain three protein-protein interaction domains: a WW domain at the N-terminal involved in interactions with proline-rich sequences and two phosphotyrosine binding domains (PTB1 and PTB2) located at the C-terminal. The second PTB domain (PTB2) is responsible for the interaction between Fe65 and the sequence 682YENPTY687 of the APP (following the numbering of the APP695 isoform). The interaction between these proteins occurs in a Tyr682 phosphorylation-independent manner [13]. The possibility of AICD to form multiprotein complexes through its association with Fe65 and its multiple ligands (Table 1) has unexpectedly expanded the potential roles of AICD.

### 2.1. Roles in APP Trafficking and Processing

AICD binds to Fe65 in a region that is essential for A*β* production, making Fe65 a good candidate for regulating APP processing. This could occur via two mutually-exclusive pathways: the amyloidogenic pathway, leading to A*β* production mediated by the *β*-secretase and the nonamyloidogenic pathway leading to the production of a large extracellular fragment (sAPP*α*), which is mediated by the *α*-secretase and prevents the generation of A*β*. Fe65 acts as a potent modulator by altering the balance between the two pathways. The overexpression of Fe65 in cell lines induces a dramatic increase in A*β* secretion [40], whereas A*β* secretion was decreased in Fe65 knockdown cells [41] and in hippocampal neurons of Fe65/Fe65L1 knockout (KO) mice [42]. The effect on the A*β* secretion appears to be dependent on the interaction between Fe65 and APP, because the knock-in mice carrying the Y682G mutation, that inhibits AICD binding to Fe65, show decreased levels of A*β* and a massive increase in sAPP*α*, as a consequence of the nonamyloidogenic pathway [43]. This is in agreement with a study showing that Fe65 is a potent suppressor of the nonamyloidogenic pathway in primate cells [44].

The mechanism by which Fe65 modulates A*β* secretion is related to its interaction with the apolipoprotein E (ApoE) receptors: the low density lipoprotein receptor-related protein (LRP) [22] and ApoE receptor 2 (ApoER2) [30].

Related to the participation of the aforementioned receptors, the effect of Fe65 in the secretion of soluble APP fragments is lost in cells lacking LRP [45]. The functional relation with ApoER2 is more complex and depends on the presence of its extracellular ligand, reelin, and its intracellular adapter, Dab-1. Reelin reduces A*β* secretion by promoting the binding of Dab1 to the APP and displacing Fe65, because they share the same binding region [46]. A decrease in Reelin expression in the entorhinal cortex (the first region of the brain where A*β* deposits can be observed), displayed in PDAPP transgenic mice (which carry human APP with mutations Swedish (swe) and Indiana) and in AD patients [47], could seriously affect the balance of Dab1 and Fe65 in their binding to AICD, increasing A*β* secretion. This has been observed in transgenic mice which lack Reelin expression (reeler) and carry the mutations swedish and arctic in APP [48].

### 2.2. Roles in Transcription

**Figure 1:**
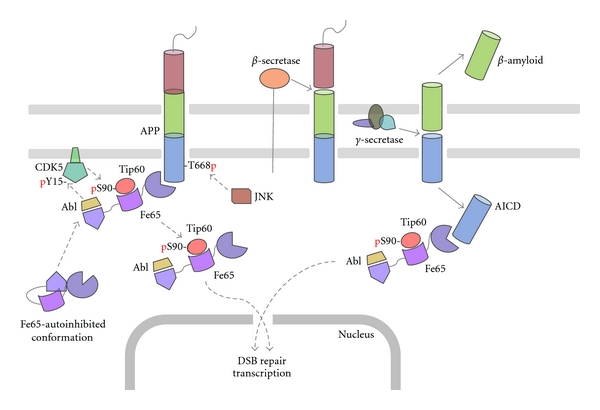
APP processing pathways involved in the activation and release of the AICD-associated complexes from the plasma membrane. Fe65 is in an autoinhibited conformation in the cytoplasm. The binding to the AICD triggers the exposure of Fe65 WW and PTB1 domains. These protein-protein domains elicit the recruitment to the subcortical domains of the plasma membrane of both c-Abl and Tip60. At the plasma membrane, c-Abl can phosphorylate and activate the protein kinase CDK-5 at Tyr15, and in turn, activated CDK-5 may phosphorylate Ser90 of Tip60. DNA damage or other unknown stimuli may then induce the release of the complex from the membrane through two complementary mechanisms: either by the activation of the *γ*-secretase or by JNK-dependent phosphorylation of Thr668 in the AICD. In spite of the preferred mechanisms involving the release of the Fe65-complex, it can be translocated to the nucleus where it activates transcription of target genes and is essential in the repair of the DNA double strand-breaks (DSB).

A decade ago, a possible role for the RIP of APP was first suggested [24]. Since APP processing seems to be similar to Notch processing, it has been suggested that RIP of APP could be involved in transcriptional regulation. In fact, the fusion of the DNA binding domain of yeast Gal4 (Gal4DB) to the C-terminal of APP induced a strong transactivation of a luciferase reporter dependent on the formation of a trimeric complex with the adapter protein Fe65 and the histone acetyltransferase Tip60 [24]. A reciprocal experiment using Tip60 or Fe65 fused to the Gal4DB gave rise to some contradictory results [49, 50]. Nevertheless, a consensus model can be generated including the vast majority of observations derived from these studies (Figure 1).

- The APP acts as an anchor for Fe65 and Fe65-associated proteins that is,: Tip60, inducing its association with membrane compartments [51]. Membrane recruitment seems to be essential for the activation of the complex, since the overexpression of soluble AICD has no effect on transactivation [49].
- The binding of APP to Fe65 induces a conformational change that “opens” the autoinhibited conformation of Fe65, produced by the association of the WW domain with a region flanked by the PTB1 and PTB2 domains [49].
- The association with the plasma membrane allows the activation of the complex, induced by the phosphorylation of Tip60 by cyclin-dependent kinases (CDKs) [52]. An excellent prospective candidate is CDK-5, that can be found associated with plasma membranes through its activator p35 and displays high activity in the brain [53].
- The release of the complex from the plasma membrane may be produced by the APP cleavage by *γ*-secretase [24] or additionally by the APP phosphorylation at Thr668 [54] which induces a conformational change in the region recognized by Fe65, decreasing the affinity for each other [55].
- Although some groups have observed AICD in the nucleus [56], particularly in nuclear domains such as transcriptional factories [57], the splicing factor compartment [58] or directly at promoters of some genes [59–61], apparently in the artificial transactivation system, the nuclear translocation of AICD is not essential to enhance luciferase expression [49].
- The N-terminal region of Fe65 that includes the WW domain is necessary for nuclear translocation [51] and therefore for its activity as a transactivating protein [24]. Although this region lacks a nuclear localization sequence (NLS), it could be directed to the nuclei by association with a protein carrying a functional NLS. A good candidate to perform this function would be the nucleosome assembly protein SET that binds the WW domain and is required for transactivation mediated by the Fe65Gal4DB fusion protein [26].
- The phosphorylation of Tyr547 in the Fe65 PTB2 domain mediated by the Abl kinase stimulates its transactivational activity [62], possibly preventing the association of Fe65 with Dexras, a Ras family GTPase, that acts as an inhibitor of the complex [34].

The search for target genes regulated by the AICD has been complex and has yielded conflicting findings. It has been reported that the AICD/Fe65 complex regulates the APP expression itself [63], glycogen synthase kinase (GSK)-3*β* [63, 64], Tip60 [63], the *β*-secretase (BACE1) [63], the primate-specific caspase 4 [35], the A*β* degrading enzyme neprylisin [61, 65, 66], the tetraspanin KAI1 [26, 63], the lipoprotein receptor-related protein (LRP1) [60], the epidermal growth factor receptor (EGFR) [67], and the tumor suppressor p53 [68]. Nevertheless, many of these studies have been refuted by others, which using different strategies for modulating the AICD/Fe65 complex did not produce changes in the expression of the aforementioned genes [69–73].

The possible origin of the reported differences is unclear, but regarding the most intensively discussed target, neprilysin, recent data may shed light on the controversy. It was shown that the AICD-binding to neprilysin gene promoter is cell type-dependent [61, 74]. Furthermore, AICD-dependent gene regulation is influenced by the passage number and cell density [75], providing two likely experimental explanations for this disagreement.

### 2.3. Roles in DNA Repair

The majority of the evidence pointing to a role of AICD in transcriptional responses derives from the use of artificial reporter systems that in fact measure the release of components from the membrane, without monitoring endogenous transcriptional activity. Besides the potential participation of Fe65 in promoting the expression of several genes described above, Fe65 has been also proposed to perform other nuclear functions such as the repair of DNA damage. Fe65 KO mice are more sensitive to DNA damage, and this can be overcome by increasing the availability of nuclear Fe65 [76]. Moreover, genotoxic damage produces a rapid translocation of Fe65 to the nuclear matrix [77] and stimulates APP processing by the *γ*-secretase complex [76] and APP phosphorylation in Thr668 [77], two mechanisms that allow translocation to the nucleus of the complexes associated with AICD. Fe65 is required for efficient repair of DNA double strand breaks (DSB), a function that depends on its interaction with Tip60 and AICD [78]. The Fe65-dependent recruitment of Tip60 to DSB sites is essential because the histone acetyltransferase activity leads to chromatin opening at the injury site, enabling the access of the complexes involved in repair [79]. On the other hand, Tip60 acetylates and activates the ataxia telangiectasia mutated (ATM) kinase [80] which in turn phosphorylates a histone H2A variant, called H2AX, which acts as a mark for the recruitment of the reparation machinery. Changes in H2AX phosphorylation could be also dependent on the stability of p53 in a mechanism that requires the accumulation of Fe65 in the nuclei [81, 82]. However, the fact that phosphorylated H2AX may be also increased in Fe65 KO cells under genotoxic damage [76] suggests that complementary mechanisms may regulate this behavior.

### 2.4. Roles in Brain Development

Fe65 is highly enriched in the brain where it is expressed as two isoforms produced by the alternative splicing of a 6 bp miniexon. The isoform that includes this exon (which encodes Arg-Glu inserted in the PTB1 domain) is expressed exclusively in neurons, whereas the isoform lacking these two aminoacids is expressed only in nonneuronal cells [83]. Fe65 protein expression may change during development [84] and also in pathological conditions such as AD [85], opening up the possibility that it participates in plastic processes in neurons, which is reflected in the phenotype of Fe65 and Fe65L1 double KO mouse. These mice exhibit defects in the positioning of cortical neurons characterized by the presence of ectopic neurons that break the pialmeningeal basement membrane and displace Cajal-Retzius neurons and also have serious defects in axonal projections [86]. Many of these phenotypical features are shared by mice lacking some of the Fe65-binding partners such as the APP family [87] and the mammalian homolog of Drosophila enabled (Mena) [88]. Mena belongs to a family of proteins that regulate actin dynamics and thereby modulate cell motility and morphology. Mena is located in areas of dynamic actin remodeling such as lamellipodia and growth cones and interacts with the actin-binding protein, profilin. Mena interacts with the Fe65 WW domain, assembling a macromolecular complex with APP [20] that regulates axonal branching [89], cell motility [90], and possibly the dynamics of actin at the growth cone and synapsis [91].

In a previous attempt to generate a Fe65 KO, it was expressed a truncated protein lacking the N-terminal domain and translated from Met261. This 60 kDa variant does not contain the WW domain and does not display the transactivation activity of the larger isoform [92]. In spite of the expression of this smaller protein, the animal shows defects in hippocampal-dependent learning and long-term potentiation (LTP) [93, 94]. However, it is difficult to assess whether these defects are due to the 97 kDa isoform loss or the appearance of this new 60 kDa isoform acting as a dominant negative protein. Behavioral studies in Fe65/Fe65L1 KO mice could help to clarify these points.

## 3. The AICD/Fe65 Transgenic Mice: New Perspectives in AD

Although the amyloid cascade hypothesis has become the mainstream in the study of AD neurobiological mechanisms, several groups have recently suggested that this should be at least reevaluated in the light of new findings [95–97]. Transgenic mice that overexpress the AICD and the adapter Fe65 in the forebrain (under the control of the CaMKII*α* promoter) [98] display several neuropathological features observed in various transgenic models and in the AD patients brains, with the exception that they do not show A*β* accumulation in the brain [11]. The expression of AICD together with Fe65 seems to be essential to induce an AD-like phenotype in the transgenic model, since a single AICD transgenic mouse developed by an independent group does not present the characteristics of the double transgenic [99], indicating that the functional relationship between both proteins, discussed in the previous sections, is indeed essential.

### 3.1. Cell Signaling Alterations

As in the brain of patients with AD and several other transgenic models used to study AD, the AICD/Fe65 mice show an increase in GSK-3*β* activity. Interestingly, the double AICD/Fe65 transgenic does not affect the GSK-3*β* mRNA or protein levels, as would be expected from a previous study which suggests that the kinase should be transcriptionally regulated by the AICD/Fe65 complex [64]. Kinase activation in the double transgenic is indeed correlated with an increase in the Tyr216 activating phosphorylation and a decrease in the Ser9 inhibitory phosphorylation [98]. A molecular explanation for this may be related with the fact that Fe65, through its WW domain, interacts and promotes GSK-3*β* phosphorylation on Tyr216 [33]. Increased GSK-3*β* activity in the AICD/Fe65 mice produces hyperphosphorylation of two direct targets: the microtubule-binding proteins, collapsin-response mediator protein (CRMP)-2 and tau [11, 98]. Increased CRMP-2 phosphorylation is also found in transgenic mice expressing mutated forms of APP and presenilin (PS)-1 and also in the cerebral cortex of AD patients. Increased CRMP-2 phosphorylation is an early event that precedes the formation of amyloid plaques and neurofibrillary tangles. Interestingly, this posttranslational modification seems to be specific for AD, since it has not been reported in other neurodegenerative conditions like the frontotemporal dementia and Pick's Disease [100, 101].

Hyperphosphorylation of tau is the initial event in the pathway to tau self-aggregation, forming the paired helical filaments (PHFs). PHFs are found at the core of the highly insoluble intraneuronal neurofibrillary tangles, one of the two neuropathological lesions (another is the senile plaques) that characterize the AD patients brains. The AICD/Fe65 mouse shares with 3xTg mice [102] the capacity to promote the formation of tau insoluble aggregates, which are not observed in most mouse models for AD [11].

### 3.2. Neuronal Activity Impairments

The AICD/Fe65 double transgenic mouse has nonconvulsive seizures with aging, abnormal electroencephalogram (EEG) spiking, and a greater sensitivity to seizures induced by kainic acid (KA) in young animals [103]. It also presents several alterations in hippocampal neural circuits, characterized by abnormal sprouting of the mossy fiber terminals with increased neuropeptide Y (NPY) expression and loss of calbindin-positive neurons [104]. Alterations in the EEGs and seizures have been observed in AD patients and in mouse models for this pathology, such as mice R1.40 (with APPswe), APPPS1, and PDAPP [105, 106].

### 3.3. Memory Deficits and Neurodegeneration

Aged AICD/Fe65 animals (>18 months) show neurodegeneration in the CA3 hippocampal area, although the defects in working memory (evaluated by the Y maze paradigm) start at a young age (8 months). Interestingly, these changes occur in the absence of increased A*β* levels [11]. Since most of the mouse models for AD are based on the expression of mutant variants of the human APP or presenilin found in cases of familiar AD, the identity of neurotoxic APP fragments has not been clearly discerned yet. Several studies have shown that A*β* deposition in senile plaques does not correlate with neuronal death and cognitive deficits present in different transgenic models [107, 108]. For example, the overexpression of wild type hAPP in mice produces memory deficits, tau hyperphosphorylation, synaptic loss, and neurodegeneration without inducing an increase in A*β* levels [109]. Surprisingly, overexpression of hAPP together with *β*-secretase in mice induces a decrease in A*β* levels and plaque deposition, but the animals suffer severe neurodegenerative disorders and learning defects [110]. In both models, an accumulation of C-terminal fragments of APP including the AICD is observed [109, 110]. Is it therefore possible that this fragment generated along with the A*β* may be responsible for the alterations in transgenic models of AD? Interestingly, the AD model termed PDAPP, when combined with a mutated form of the AICD (D664A), shows a complete reversion of the neuropathological hallmarks of the disease, including synaptic loss, the dentate gyrus atrophy, the astrogliosis, the deficits in synaptic transmission and memory, and the behavioral abnormalities without affecting the A*β* levels or the plaque accumulation [111–114]. These results strongly suggest that the causal relationship between the A*β* accumulation and the neuropathological defects usually associated with AD may be challenged and position the AICD as a good candidate to explain the effects observed in various transgenic models based on mutations in APP and PS1.

## 4. Conclusions

The two hallmarks of AD, the amyloid plaques, and neurofibrillary tangles, which are elegantly related through the amyloid cascade hypothesis, are the main components in the current research on the molecular mechanisms leading to this pathology. Since its origin, the amyloid cascade hypothesis has accumulated substantial evidence in its support, which has virtually overshadowed the fact that clinical trials based on this hypothesis have been shown to be unsuccessful [115]. One of many possibilities to explain the failure of clinical trials could be related with the fact that several mouse models express the human-mutated APP found in familial AD, so it is unclear which abnormalities detected in these models are product of specific A*β* species (like oligomers) or another toxic metabolites of APP (like AICD) or simply due to effects of overexpression of hAPP. However, the evidence collected from the transgenic models here reviewed could help to discern whether the A*β* species or the AICD are the key elements triggering neurodegeneration. Three independent transgenic mice lines (a single transgenic of hAPP, a double AICD/Fe65 transgenic, and the double hAPP/*β*-secretase transgenic) recapitulate the neuropathological alterations of the disease without any increase in A*β* secretion. All of these models have an accumulation of the APP C-terminal fragments. Moreover, the introduction of a point mutation in the AICD in transgenic mice expressing the hAPP with the swe and Indiana mutations, the AD-like phenotype is reversed, in spite of increased A*β* production. All of these evidences suggest that the AICD could be acting as the bona fide toxic intermediate in the AD progression and could become a target for future therapeutic interventions against this devastating disease.

## Figures and Tables

**Table 1:** Fe65 interactors and its functions.

Protein	Domain involved	Putative functions of the interaction	References
Amyloid precursor protein (APP)	PTB2	Regulation of A*β* secretion, nuclear signaling, and cytoskeleton regulation	[17]

Mammalian *enabled *(Mena)	WW	Actin polimerization	[20]

CP2/LSF/LBP1	PTB1	Transcriptional regulation, GSK-3*β* expression	[21]

Low-density lipoprotein receptor-related protein (LRP1)	PTB1	APP trafficking, A*β* secretion	[22]

Abl tyrosine kinase	WW	Nuclear signaling	[23]

Tat-interacting protein 60 kDa (Tip60)	PTB1	Nuclear signaling, DNA repair	[24]

Alcadein	ND	APP metabolism	[25]

Nucleosome assembly factor SET	WW	Transcriptional regulation	[26]

Tau	PTB1	Cytoskeleton regulation	[27]

14-3-3**γ**	Between WW and PTB1	Nuclear signaling	[28]

P2X receptor	WW	Synaptic transmission	[29]

ApoER2	PTB1	APP trafficking, A*β* secretion	[30]

Estrogen receptor *α*	ND	Transcriptional regulation	[31]

NIMA-related kinase 6	WW	Apoptosis	[32]

Glycogen synthase kinase-3**β**	WW	Kinase activation	[33]

Dexras1	PTB2	Nuclear signaling	[34]

Teashirt	PTB1	Repression of caspase 4 expression	[35]

Neuronal precursor cell expressed developmentally down regulated 4-2 (Nedd 4-2)	WW	Fe65 ubiquitylation	[36]

Dab1	ND	APP processing	[37]

Megalin	ND	Axonal branching, APP trafficking	[38]

Rac1	ND	Fe65 expression	[39]

ND: Not determined
